# Surgical Impact on Circulating Tumor and Hybrid Cells in Head and Neck Cancer: A Pilot Study

**DOI:** 10.32604/or.2026.076831

**Published:** 2026-10-28

**Authors:** Evgeniya S. Grigoryeva, Anna Y. Kalinchuk, Irina K. Fedorova, Vladislav O. Tskhai, Denis E. Kulbakin, Liubov A. Tashireva

**Affiliations:** 1 The Laboratory of Molecular Therapy of Cancer, Cancer Research Institute, Tomsk National Research Medical Center, Russian Academy of Sciences, Tomsk, Russia; 2 The Department of Head and Neck Cancer, Cancer Research Institute, Tomsk National Research Medical Center, Russian Academy of Sciences, Tomsk, Russia

**Keywords:** Circulating tumor cells, surgical intervention, head and neck cancer, recurrence-free survival

## Abstract

**Objective:** Surgery is known to significantly influence both the quantity and functional characteristics of circulating tumor cells (CTCs) in the bloodstream. Our cohort study aimed to characterize the landscape of CTCs and circulating hybrid cells (CHCs) in patients with head and neck cancer in peri-operative period. **Methods:** 26 patients were recruited and CTC and CHC counts were evaluated using four epithelial markers (EpCAM with differential localization of expression—membrane and intracellular, cytokeratin 7/8, and pan-cytokeratin) by flow cytometry. Expression of EpCAM and pan-cytokeratin in tumor tissue slides was assessed by multiplex immunofluorescence analysis. **Results:** The study revealed that CD45^+^ CHCs dominated the circulating landscape, outnumbering classical CD45^−^ CTCs by over forty-fold. Both compartments exhibited phenotypic heterogeneity reflecting ongoing epithelial-to-mesenchymal transition. Surgical resection significantly depleted specific CD45^+^ CHC subpopulations but had no effect on CD45^−^ CTCs. Postoperative CHC counts above 2.5 cells/mL were associated with favorable outcomes, whereas depletion below this threshold predicted poor clinical outcomes and relapse. Although the patient cohort was limited, robust statistical analyses validate the significance of our observations. **Conclusion**: Our findings identify a novel informative marker for predicting head and neck cancer progression, which may serve as part of a non-invasive liquid biopsy.

## 1 Introduction

Surgical removal of solid tumors remains the primary treatment method for cancer, enabling a cure in localized forms of the disease. However, accumulating evidence suggests that the process of tumor resection itself can trigger complex biological responses that influence disease progression [1,2,3]. Circulating tumor cells (CTCs) are considered a key component of the metastatic cascade and a valuable biomarker in oncology, particularly in the context of liquid biopsy [4,5,6]. In squamous cell carcinoma of the oral cavity and other head and neck cancers, their detection correlates with poor prognosis, including an increased risk of recurrence and distant metastases [7,8,9,10]. Despite their extremely low concentration in the blood, CTCs provide valuable insights into the biological behavior of tumors that are not accessible through conventional TNM staging [11]. This is especially important considering that the 5-year survival rate for SCCOC remains at 50–60% and has shown little improvement in recent years [12]. 

Due to the absence of a gold standard protocol, the sensitivity and specificity of CTC enumeration methods vary considerably [13,14,15,16]. Contemporary research emphasizes the necessity of evaluating CTCs while considering their phenotypic heterogeneity. Traditionally, CTC detection has relied on EpCAM and cytokeratins co-expression [17], however, accumulating evidence shows that during epithelial-mesenchymal transition (EMT), tumor cells lose certain markers and begin to express mesenchymal markers [18]. Thus, the use of combined marker panels significantly enhances sensitivity and reflects the true diversity of CTCs in cancer patients’ blood. In patients with HNC, the detection of hybrid EMT phenotypes of cells is associated with a higher likelihood of metastasis and poorer clinical outcomes [19].

Of particular interest are circulating hybrid cells (CHCs), which simultaneously express epithelial markers (EpCAM and cytokeratins) and the pan-leukocyte marker (CD45). This dual expression renders them more resistant to immune surveillance and endows them with high metastatic potential [20]. Studies indicate that CHCs may constitute a majority of all tumor cells in circulation and serve as an independent adverse prognostic factor in various cancer types [21]. However, there is limited data available on CHCs in head and neck cancer [22].

Overall, key questions remain unresolved regarding the diversity of CTCs and CHCs, the impact of surgical intervention on their profiles, and their association with the clinical course of head and neck cancer. Our study demonstrates significant heterogeneity among CD45^−^ CTCs and CD45^+^ CHCs in the peri-operative period, revealing changes in their subpopulation dynamics that correlate with clinical parameters. The null hypothesis—that surgical intervention does not affect the composition or abundance of these circulating cell populations—was tested. Addressing this hypothesis, our findings highlight the potential of liquid biopsy methods to monitor residual tumor cell dynamics. This paves the way for precision surgical oncology, where treatment strategies consider not only the primary tumor but also the behavior of residual cellular populations, potentially improving therapeutic outcomes. Although conducted on a limited patient cohort, the use of robust statistical methods supports the significance of our results. The primary aim of this study was to characterize the landscape of CD45^−^ CTCs and CD45^+^ CHCs in patients with head and neck cancer during the peri-operative period.

## 2 Material and Methods.

### 2.1 Patients

The prospective study enrolled 26 patients diagnosed with head and neck squamous cell carcinoma ranging from stage T1–4N0–3M0, who were admitted for treatment at the Cancer Research Institute, Tomsk NRMC (Table 1), as well as 9 healthy female donors matched by age. The study received approval from the local ethics committee of the Tomsk NRMC Institutional Review Board on June 17, 2016 (approval No. 8) and all patients provided informed consent before participating in the study. The study was conducted in accordance with the Declaration of Helsinki. Patients received treatment in accordance with the NCCN Clinical Practice Guidelines [23]. The follow-up period was 15 months, during which four patients developed distant metastases.

**Table 1:** Clinicopathological characteristics of head and neck cancer patients.

Parameter	Characteristics	Frequency, % (n/N)
Age, years	≤50	19.23% (5/26)
	>50	80.77% (21/26)
Tumor size (T)	1	15.38% (4/26)
	2	19.24% (5/26)
	3	50.00% (13/26)
	4	15.38% (4/26)
Lymph node status (N)	0	57.69% (15/26)
	1	30.77% (8/26)
	2	3.85% (1/26)
	3	7.69% (2/26)
Localization	tongue	34.62% (9/26)
	alveolar process of the mandible	30.77% (8/26)
	oral mucosa	30.77% (8/26)
	lateral surface of the tongue	3.84% (1/26)

Note: n: sample numbers; N: total sample numbers (26).

Inclusion criteria were as follows: ability to test blood samples prior to treatment; patients’ general condition with an ECOG–WHO performance status of 0–2; absence of significant somatic contraindications to surgery; morphological and immunohistochemical confirmation of invasive carcinoma of no special type (ISC) of any molecular subtype.

### 2.2 Flow Cytometry

Venous blood samples in EDTA tubes were obtained before surgery and 72 h postoperatively. For flow cytometry, 3 mL aliquots were subjected to 1-h incubation at 37°C. After incubation, the plasma and leukocyte layers were removed following red blood cell sedimentation. Cell pellets were resuspended and washed in 2 mL Cell Wash buffer (#349524, BD Biosciences, San Jose, CA, USA) by centrifugation (800× *g*, 15 min, room temperature (RT)).

Immunophenotyping of CTCs followed our previously published protocol [24]. Initial labeling targeted surface antigens (CD45 and EpCAM), while intracellular markers were detected in a second staining phase. Samples were first treated with 5 μL Fc Receptor-Blocking Solution (#2711505, Human TruStain FcX, Sony Biotechnology, San Jose, CA, USA) for 10 min at RT to prevent non-specific binding. Cells were then labeled with the following surface antibodies for 20 min at RT: BV570-conjugated anti-CD45 (clone HI30, Sony Biotechnology) and APC/Fire750-conjugated anti-EpCAM (clone 24D2, Biolegend, San Diego, CA, USA). Erythrocytes were removed by adding 250 μL OptiLyse C lysis buffer (Beckman Coulter, Villepinte, France) and incubating for 10 min at RT in the dark. The resulting suspension was pelleted and washed in 1 mL Cell Wash buffer (BD Biosciences) by centrifugation (800× *g*, 6 min, RT).

To detect intracellular epithelial markers (EpCAM, CK, panCK), cells were fixed and permeabilized with 250 μL BD Cytofix/Cytoperm solution (#554722, BD Biosciences) for 20 min at 4°C in darkness, followed by two washes in 1 mL BD Perm/Wash buffer (#554723, BD Biosciences) via centrifugation (800× *g*, 6 min, RT). Cell pellets were resuspended in 50 μL BD Perm/Wash buffer (BD Biosciences) and incubated for 10 min at 4°C in the dark. Samples were first treated with 5 μL Fc Receptor-Blocking Solution (#2711505, Human TruStain FcX, Sony Biotechnology) for 10 min at RT to prevent non-specific binding. The following intracellular antibodies were then added according to the manufacturer’s recommendations and incubated for 20 min at 4°C: BV 605-conjugated anti-EpCAM (clone 9C4, Sony Biotechnology), PE-CF594-conjugated anti-Cytokeratin (clone CAM5.2, BD Biosciences), and eFluor 660-conjugated anti-pan Cytokeratin (panCK) (clone AE1/AE3, eBioscience, Frankfurt, Germany). Following antibody incubation, samples underwent a final wash in 1 mL Cell Wash buffer (#349524, BD Biosciences) by centrifugation (800× *g*, 6 min, RT) before resuspension in 100 μL Stain buffer (Sony Biotechnology).

Quality control measures included unstained samples and antibody-stained controls. Isotype control samples received matching concentrations of corresponding isotype antibodies. Fluorescence compensation was established using VersaComp Antibody Capture Beads (Beckman Coulter). Samples were analyzed on a Novocyte 3000 flow cytometer (ACEA Biosciences, San Diego, CA, USA) using NovoExpress 1.3.0 software (Agilent Technologies, Santa Clara, CA, USA). Cell populations were identified through sequential gating: initial selection excluded debris and doublet events based on forward scatter (FSC) and side scatter (SSC) characteristics, followed by separation into CD45^−^ (CTCs) and CD45^+^ (CHCs) populations (Fig. A1. The gating strategy of CD45^−^ CTCs and CD45^+^ CHCs in patients with head and neck cancer). The flow cytometry data in the Results section are presented as median with interquartile range (IQR).

### 2.3 Multiplex Immunofluorescence Analysis

Multiplex immunofluorescence staining was performed on 4-μm formalin-fixed, paraffin-embedded tissue sections using a BOND RXm automated stainer (Leica, HIS2330, Nussloch, Germany). The staining protocol was based on sequential cycles of tyramide signal amplification (TSA). Each complete cycle for a single marker included the following steps: heat-induced epitope retrieval in Epitope Retrieval Solution 2 buffer (Leica, AR9640) at 98.5°C for 20 min; incubation with EnVision FLEX Peroxidase-Blocking Reagent (Agilent, SM801, Santa Clara, CA, USA) for 10 min; incubation with Novocastra Protein Block (Leica, RE7102) for 10 min; incubation with the primary antibody for 25 min at room temperature; detection with an appropriate HRP-conjugated secondary antibody (Leica) for 20 min at room temperature; and signal amplification with a tyramide-fluorophore conjugate for 20 min at room temperature. Between each sequential TSA cycle, an additional heat-induced epitope retrieval step was performed to denature antibodies from the previous round and prevent cross-reactivity.

The following panel of primary antibodies was used: anti-pancytokeratin (clone AE1/AE3; Dako, M3515, Glostrup, Denmark; 1:5), anti-EpCAM (clone E144; Abcam, ab32392, Cambridge, UK; 1:1000), and anti-CD45 (Leica; 1:5). Signal amplification was carried out using tyramide conjugates diluted 1:350: Alexa Fluor 647 for pancytokeratin, Alexa Fluor 488 for EpCAM, and Alexa Fluor 555 for CD45.

Following the final TSA cycle, cell nuclei were counterstained with DAPI (Sigma-Aldrich, MBD0015, St. Louis, MO, USA). Slides were mounted in mounting medium with DAPI and scanned using the Vectra 3.0 system (PerkinElmer, CLS143047, Shelton, CT, USA). Images were processed using inForm 2.2.1 software (PerkinElmer). The proportions of different tumor cell populations were quantified as percentages of all tumor cells.

### 2.4 Statistical Analyses

Data analysis was performed using the GraphPad Prism 10 (GraphPad Software, San Diego, CA, USA). Differences between independent groups were evaluated using the Mann–Whitney U test and Kruskal–Wallis test. The Wilcoxon matched-pairs signed rank and Friedman tests were used for comparisons between dependent groups; the sample was also checked for outliers. Recurrence-free survival was assessed with a univariate Cox regression model, which yielded hazard ratios (HRs). This model adjusted for stage T and N of the primary tumor and CHC count. Recurrence-free survival was calculated by the Kaplan–Meier method. Correlation analysis was performed using Spearman’s rank correlation test. A *p*-value < 0.05 was considered statistically significant and is indicated in red throughout all figures. 

## 3 Results

### 3.1 Landscape of Circulating Tumor Cells (CTCs) and Circulating Hybrid Cells (CHCs) in Patients with Head and Neck Cancer Prior to Surgery

In this study, cells positively expressing epithelial markers (EpCAM and/or cytokeratins) but lacking CD45 were defined as CTCs; conversely, cells co-expressing CD45 and these epithelial markers were defined as CHCs. The epithelial origin was confirmed using both membrane (EpCAM(m)) and intracellular (EpCAM(ic)) expression of EpCAM, as well as cytokeratin 7/8 (CK7/8), and pan-cytokeratin (panCK). Utilizing this extended panel of four epithelial markers allows for maximized detection sensitivity for heterogeneous CTC/CHC populations. Detection of cytokeratins allows identification of tumor cells lacking EpCAM expression, which are known to arise during the epithelial-mesenchymal transition process. Thus, CTCs and CHCs were required to express at least one of the indicated epithelial markers. In 24 out of 26 patients, CTCs were detected in the blood prior to surgical treatment, while CHCs were found in all patients. The data demonstrate that in patients with head and neck cancer, CHCs quantitatively predominate over CTCs (170.50 (79.00–557.88) cells/mL versus 4.0 (2.0–7.13) cells/mL, *p* < 0.0001). The median count of hybrid cells (170.5 cells/mL) exceeded the median count of CTCs (4.0 cells/mL) by 42.6-fold.

Using an extended panel of four epithelial markers, we were able to assess the heterogeneity of CTCs and CHCs in 26 patients with head and neck cancer. CD45^−^ EpCAM(m)^+^ EpCAM(ic)^+^ CTCs represent cells with the most classical CTC phenotype; however, these cells are detected least frequently and in the lowest numbers in patient blood (4.0%, 0.00 (0.00–0.00) cells/mL). The population of CD45^−^EpCAM(m)^−^EpCAM(ic)^−^ CTCs corresponds to a pool of cells exhibiting EMT features, occurring frequently and in substantial numbers (80.0%, 3.00 (1.00–4.75) cells/mL). The CD45^−^EpCAM(m)^−^EpCAM(ic)^+^ CTC subgroup also displays EMT characteristics, having lost membrane EpCAM expression, which may signify initiation of the EMT program. This population was detected in 52% of patients (0.50 (0.00–1.25) cells/mL). The CD45^−^EpCAM(m)^+^EpCAM(ic)^−^ CTC subpopulation constitutes an unexpected finding, since EpCAM is primarily considered a membrane protein with an intracellular domain; absence of intracellular domain detection despite the presence of membrane EpCAM may indicate peculiarities in the binding sites of the antibodies used.

Specifically, the CD45^−^EpCAM(m)^−^EpCAM(ic)^−^ CTCs comprised cells with comparable expression levels of cytokeratins 7/8 and pan-cytokeratins (Fig. 1A). The median counts of CK7/8^+^panCK^−^ and CK7/8^−^panCK^+^ subpopulations were 1.00 (0.00–4.25) cells/mL and 0.00 (0.00–1.25) cells/mL, respectively. In contrast, double-positive CK7/8^+^ panCK^+^ CTCs were almost absent within the CD45^−^EpCAM(m)^−^EpCAM(ic)^−^ subpopulation, with the lowest median count of 0.00 (0.00–0.00) cells/mL, significantly lower than the CK7/8^+^panCK^−^ and CK7/8^−^panCK^+^ subpopulations (*p* = 0.0002 and *p* = 0.0184, respectively).

In contrast, the CD45^−^EpCAM(m)^−^EpCAM(ic)^+^ CTCs were predominantly composed of cells lacking cytokeratin expression (*p* = 0.0214 and *p* = 0.0137), potentially indicating an advanced stage of EMT (Fig. 1B). The median count of CK7/8^+^panCK^−^, CK7/8^−^panCK^+^, CK7/8^+^panCK^+^, and CK7/8^−^panCK^−^ cells are represented in Table 2.

The CD45^−^EpCAM(m)^+^EpCAM(ic)^−^ subpopulation, meanwhile, exhibited pan-cytokeratin expression but lacked cytokeratins 7 and 8 (Fig. 1C). Most cells exhibited the CK7/8-panCK+ phenotype (*p* = 0.0052 and *p* = 0.0044). In the CD45^−^EpCAM(m)^−^EpCAM(ic)^+^ population of a single patient, one CTC per milliliter of peripheral blood was detected, which was positive for both pan-cytokeratin and cytokeratins 7/8 (Fig. 1D).

The patterns of heterogeneity between CTCs and CHCs were similar (Fig. 2); however, the count of CHCs was significantly higher than that of CTCs across all subsets (*p* < 0.0001), except in the EpCAM(m)^+^EpCAM(ic)^+^ subset (*p* = 0.133) (Table 2). 

**Figure 1:**
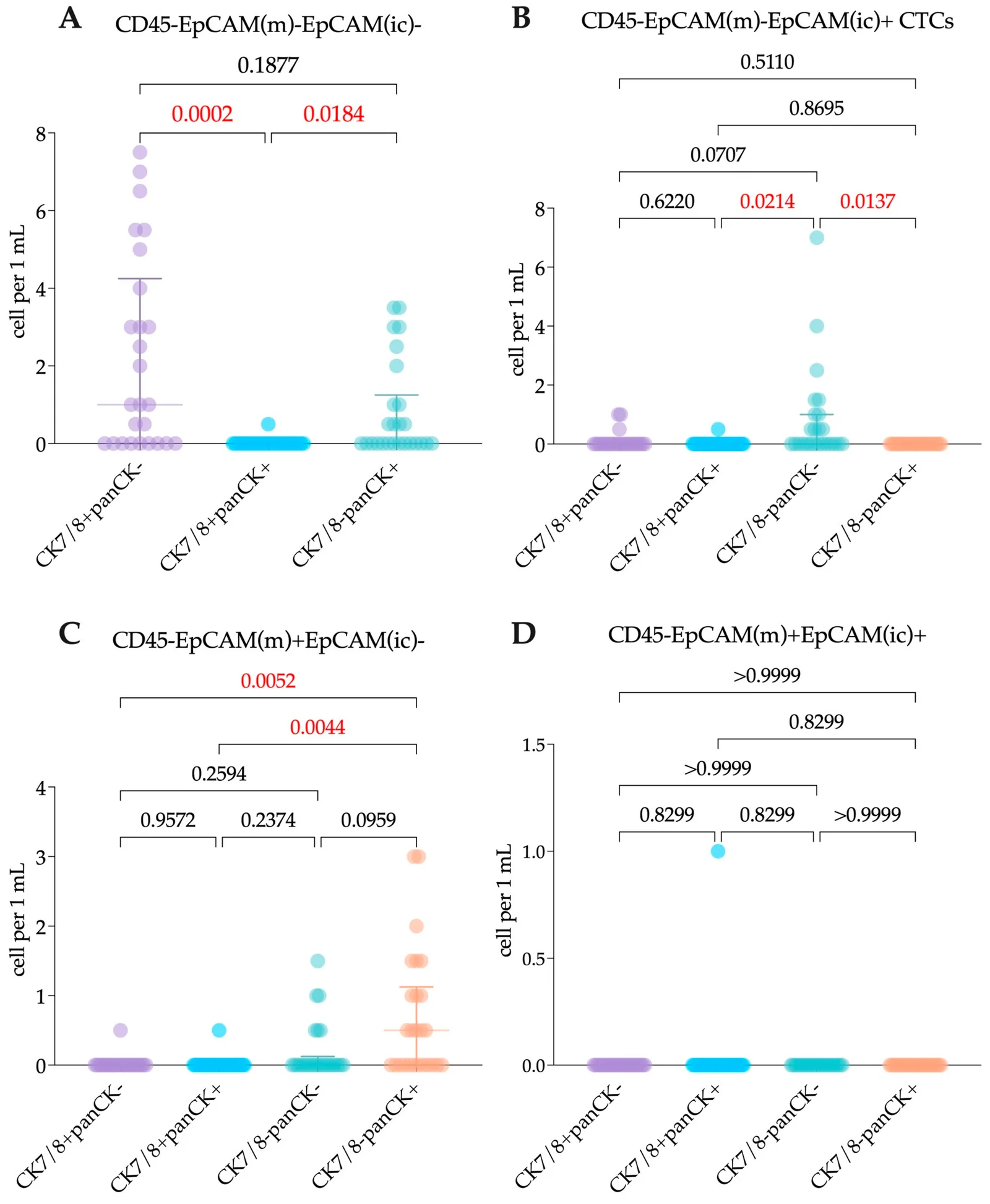
The number of CD45^−^EpCAM(m)^−^EpCAM(ic)^−^ (**A**), CD45^−^EpCAM(m)^−^EpCAM(ic)^+^ (**B**), CD45^−^EpCAM(m)^+^EpCAM(ic)^−^ (**C**) and CD45^−^EpCAM(m)^+^EpCAM(ic)^+^ (**D**) circulating tumor cells (CTCs) in head and neck cancer patients before surgery. The Friedman test was employed to compare CTC subpopulations and evaluate *p*-values in patients with head and neck cancer.

**Figure 2:**
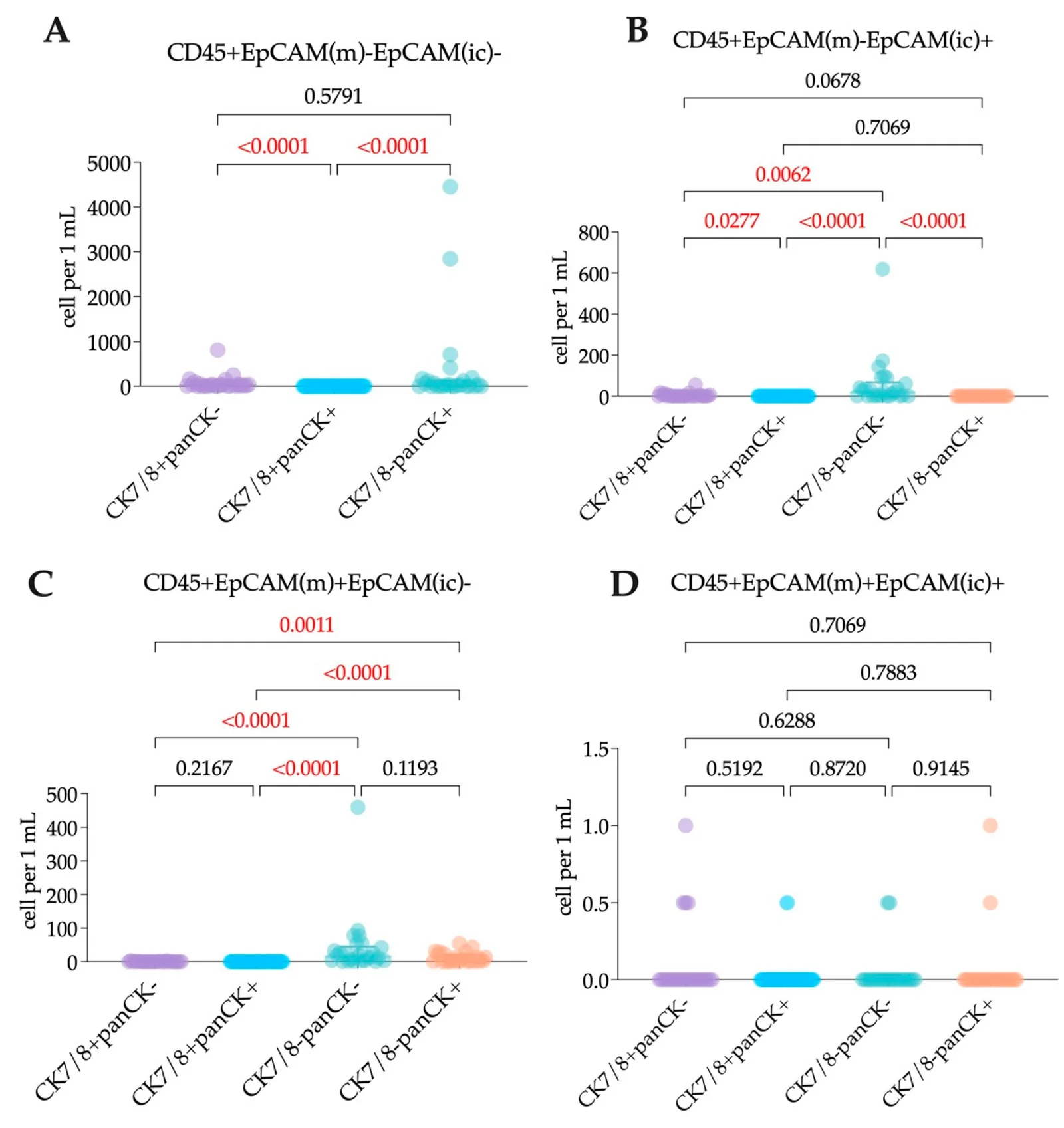
The number of CD45^+^EpCAM(m)^−^EpCAM(ic)^−^ (**A**), CD45^+^EpCAM(m)^−^EpCAM(ic)^+^ (**B**), CD45^+^EpCAM(m)^+^EpCAM(ic)^−^ (**C**) and CD45^+^EpCAM(m)^+^EpCAM(ic)^+^ (**D**) in head and neck cancer patients before surgery. The Friedman test was employed to compare CHC subpopulations and evaluate *p*-values in patients with head and neck cancer.

**Table 2:** The CTCs and CHCs counts in peripheral blood of head and neck cancer patients.

Cell Phenotype	CTCs, Cell/mL	CHCs, Cell/mL	*p*-Value
EpCAM(m)^−^EpCAM(ic)^−^, Median (range)	3.00 (0.75−4.38)	70.25 (26.50−246.8)	*p* < 0.0001
CK7/8^+^panCK^−^, Median (range)	1.00 (0.00−4.25)	26.25 (7.50−45.25)	
CK7/8^+^panCK^+^, Median (range)	0.00 (0.00−0.00)	0.00 (0.00−0.13)	
CK7/8^−^panCK^+^, Median (range)	0.00 (0.00−1.25)	26.50 (0.00−129.05)	
EpCAM(m)^−^EpCAM(ic)^+^, Median (range)	0.25 (0.00−1.13)	20.0 (1.25−79.00)	*p* < 0.0001
CK7/8^+^panCK^−^, Median (range)	0.00 (0.00−0.00)	0.00 (0.00−6.00)	
CK7/8^+^panCK^+^, Median (range)	0.00 (0.00−0.00)	0.00 (0.00−0.00)	
CK7/8^−^panCK^−^, Median (range)	0.00 (0.00−1.00)	19.75 (0.38−68.13)	
CK7/8^−^panCK^+^, Median (range)	0.00 (0.00−0.00)	0.00 (0.00−0.00)	
EpCAM(m)^+^EpCAM(ic)^−^, Median (range)	0.75 (0.00−1.50)	34.00 (8.75−67.38)	*p* < 0.0001
CK7/8^+^panCK^−^, Median (range)	0.00 (0.00−0.00)	0.00 (0.00−1.00)	
CK7/8^+^panCK^+^, Median (range)	0.00 (0.00−0.00)	0.00 (0.00−0.00)	
CK7/8^−^panCK^−^, Median (range)	0.00 (0.00−0.13)	15.15 (3.38−44.75)	
CK7/8^−^panCK^+^, Median (range)	0.50 (0.00−1.13)	5.00 (0.88−21.88)	
EpCAM(m)^+^EpCAM(ic)^+^, Median (range)	0.00 (0.00−0.00)	0.00 (0.00−0.50)	*p* = 0.133
CK7/8^+^panCK^−^, Median (range)	0.00 (0.00−0.00)	0.00 (0.00−0.00)	
CK7/8^+^panCK^+^, Median (range)	0.00 (0.00−0.00)	0.00 (0.00−0.00)	
CK7/8^−^panCK^−^, Median (range)	0.00 (0.00−0.00)	0.00 (0.00−0.00)	
CK7/8^−^panCK^+^, Median (range)	0.00 (0.00−0.00)	0.00 (0.00−0.00)	

Note: CTCs: circulating tumor cells, CHCs: circulating hybrid cells.

This observation may indirectly support a tumor origin for a portion of the CHCs. All patients exhibited populations of CD45^+^EpCAM(m)^−^EpCAM(ic)^−^ and CD45^+^EpCAM(m)^+^EpCAM(ic)^−^ cells. The CD45^+^EpCAM(m)^−^EpCAM(ic)^−^ CHCs were mostly CK7/8^+^panCK^−^ or CK7/8^−^panCK^+^, but not CK7/8^+^panCK^+^. In contrast, the CD45^+^EpCAM(m)^+^EpCAM(ic)^−^ population was predominantly composed of CK7/8^−^panCK^−^ cells. Interestingly, the CD45^+^EpCAM(m)^−^EpCAM(ic)^+^ population was also mainly represented by CK7/8^−^panCK^−^ cells (Table 2).

### 3.2 Changes in the CTC and CHCs Landscape in Patients with Head and Neck Cancer Undergoing Surgery

Post-surgery, a significant reduction in the overall count was observed only in the CHC population. Specifically, the number of CHCs decreased from 172.5 (82.0–564.3) to 93.0 (29.75–164.25) cells/mL (*p* = 0.013). The median CTC count pre- and post-surgery was 4.00 (2.25–7.75) cells/mL and 2.50 (0.75–6.50) cells/mL, respectively (*p* = 0.426). Post-surgery, individual-level observations revealed varied responses in the total count and specific subpopulations of CTCs. Specifically, 12 out of 21 patients showed a decrease, 1 patient showed no change, and 8 patients exhibited an increase in total CTC count. Blood samples from 5 out of 26 patients lacked a second time point analysis due to technical reasons.

Next, we evaluated how the numbers of CTC and CHC changes post-surgery, taking into account the diversity of epithelial markers expressed by these cells. No statistically significant changes were observed in any subpopulation of CTCs (*p* > 0.05) (Fig. 3).

**Figure 3:**
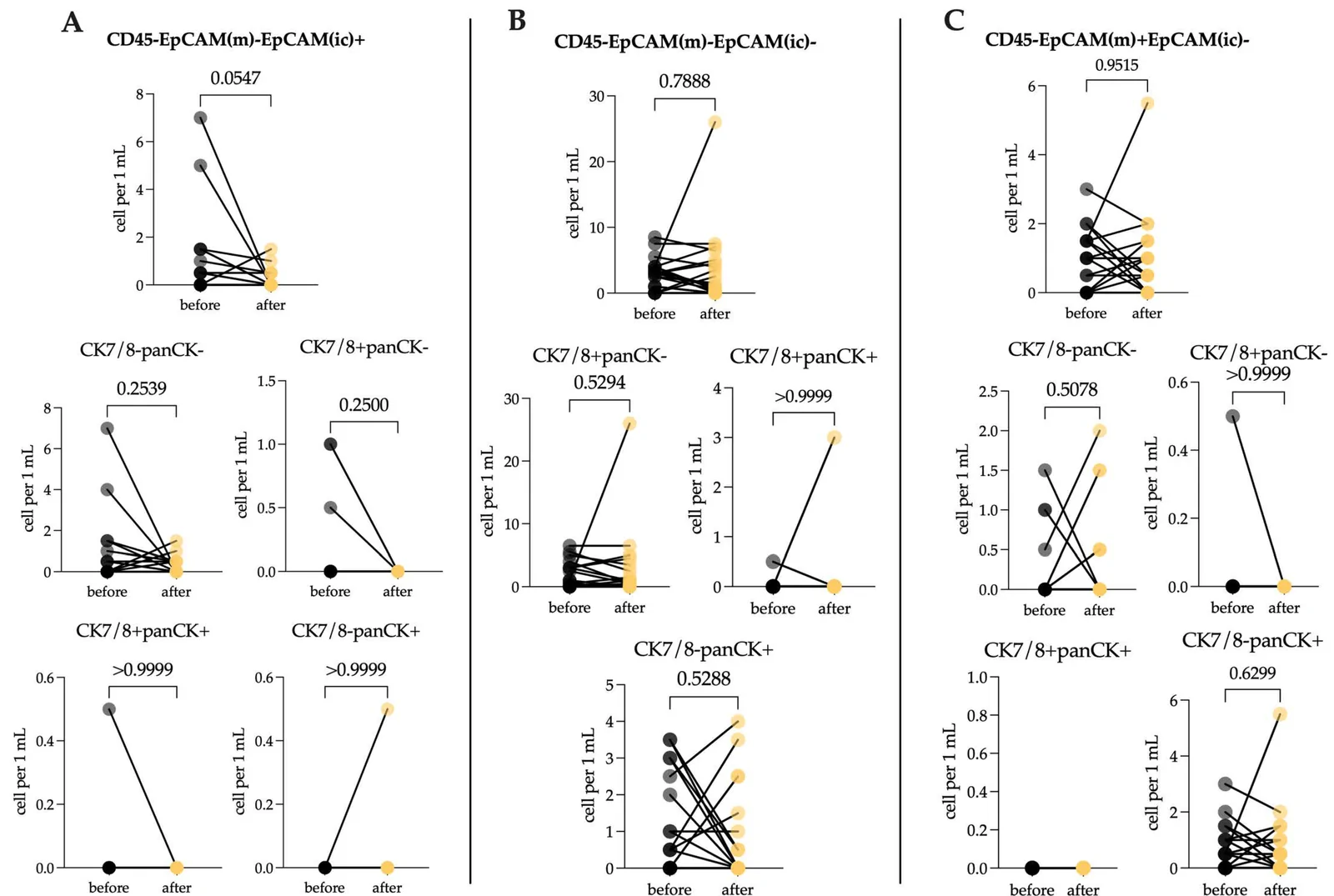
Changes in the CD45^−^EpCAM(m)^−^EpCAM(ic)^+^ (**A**), CD45^−^EpCAM(m)^−^EpCAM(ic)^−^ (**B**), CD45^−^EpCAM(m)^+^EpCAM(ic)^−^ (**C**), CTC subpopulations with EpCAM membrane and intracellular expression (**A**–**C**) in patients with head and neck cancer undergoing surgery. The Wilcoxon test was employed to compare CTC subpopulations and evaluate *p*-values in patients with head and neck cancer.

The number of cells in the two CHC subpopulations changed significantly (Fig. 4 and Fig. 5). 

**Figure 4:**
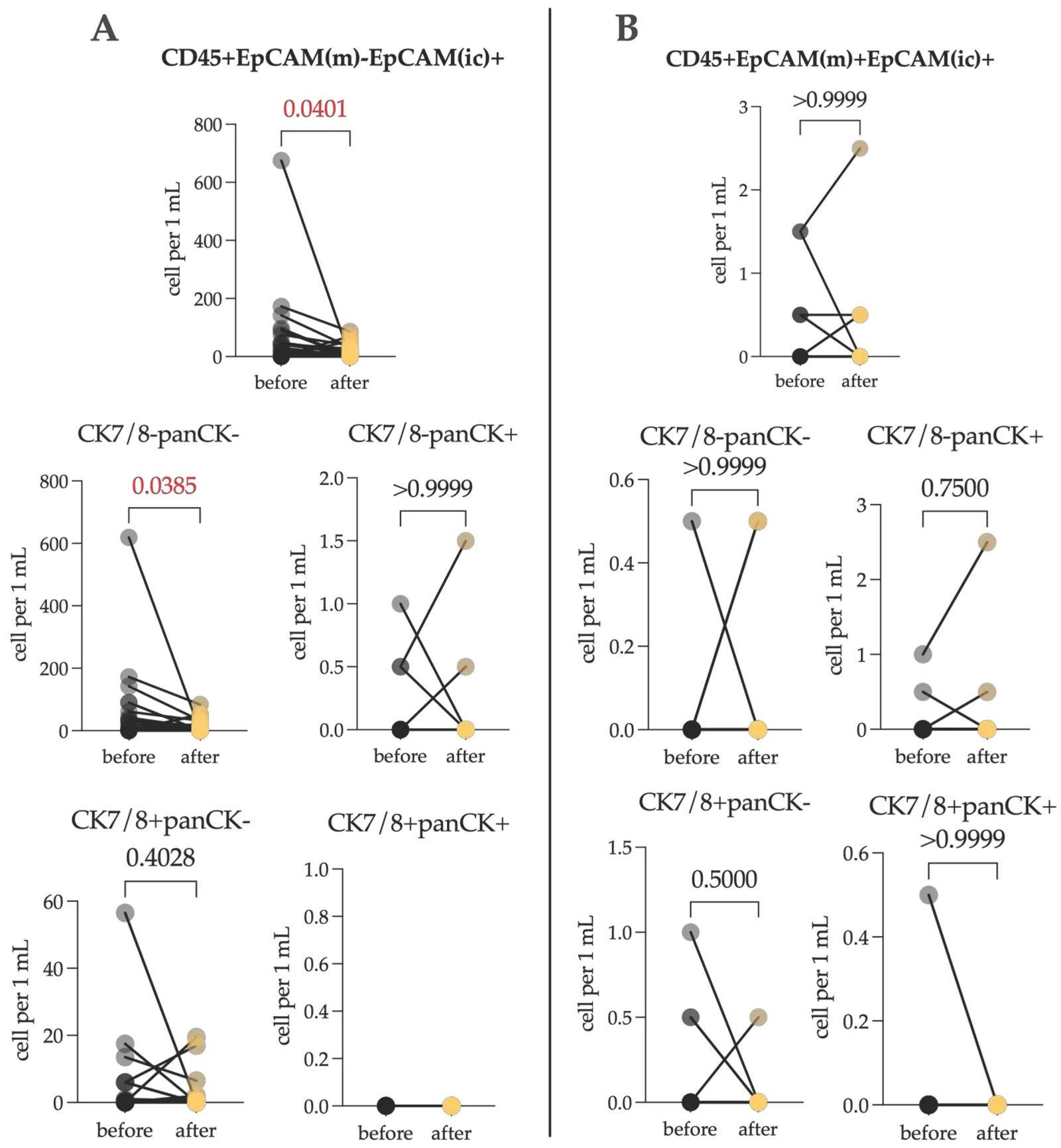
The changes in the CD45^+^EpCAM(m)^−^EpCAM(ic)^+^ (**A**) and CD45^+^EpCAM(m)^+^EpCAM(ic)^+^ (**B**) CHCs considering cytokeratins 7/8 and pan-cytokeratin expression in patients with head and neck cancer undergoing surgery. The Wilcoxon test was employed to compare CHC subpopulations and evaluate *p*-values in patients with head and neck cancer.

Notably, the counts of CD45^+^EpCAM(m)^−^EpCAM(t)^+^CK7/8^−^panCK^−^ and CD45^+^EpCAM(m)^+^EpCAM(ic)^−^CK7/8^+^panCK^−^ CHCs were significantly reduced after surgery (*p* = 0.0385 and *p* = 0.0498, respectively) (Fig. 4A and Fig. 5B). 

**Figure 5:**
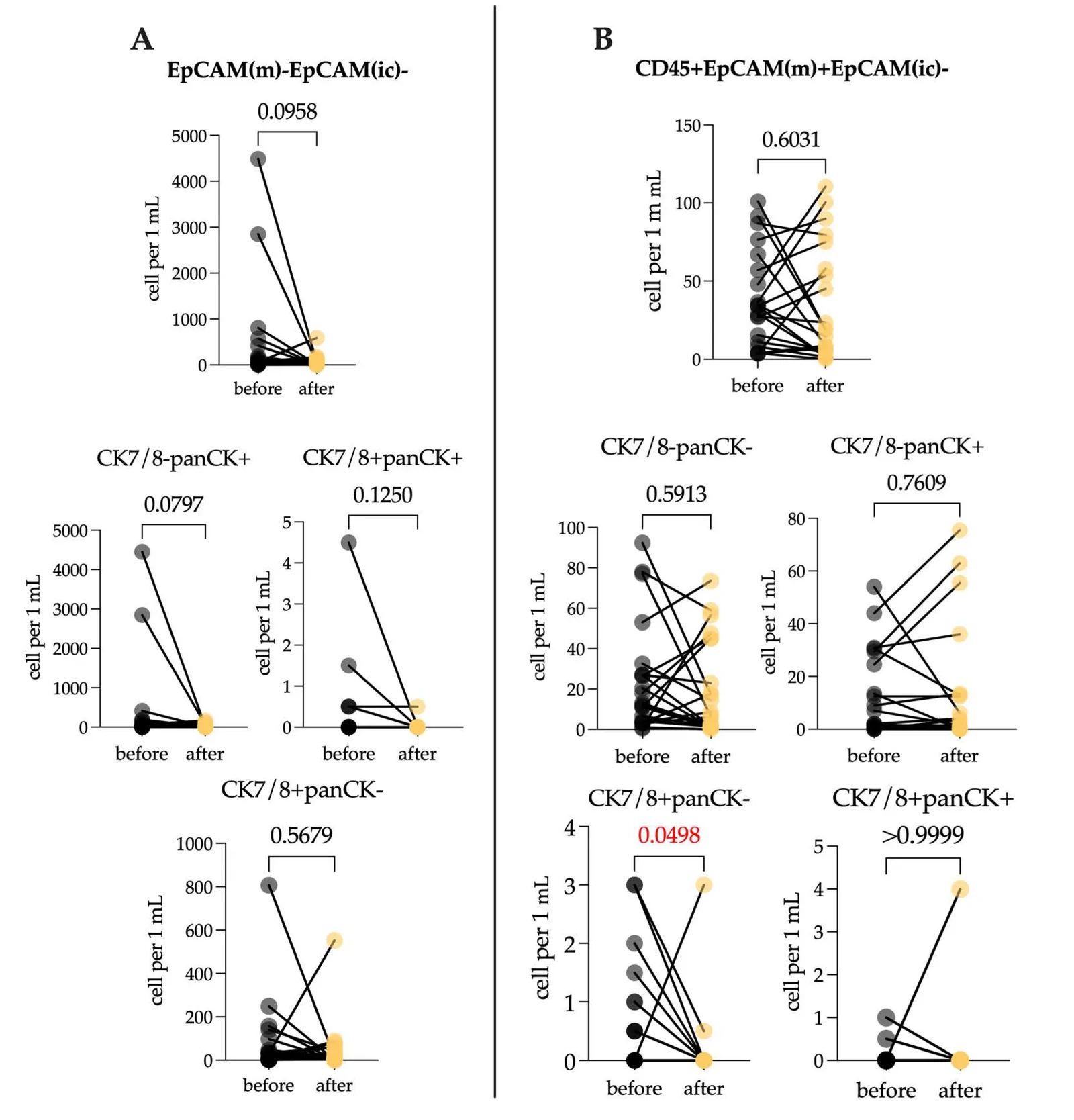
The changes in the CD45^+^EpCAM(m)^−^EpCAM(ic)^−^ (**A**) and CD45^+^EpCAM(m)^+^EpCAM(ic)^−^ (**B**) CHCs considering cytokeratins 7/8 and pan-cytokeratin expression in patients with head and neck cancer undergoing surgery. The Wilcoxon test was employed to compare CHC subpopulations and evaluate *p*-values in patients with head and neck cancer.

Before surgery, the median counts of CD45^+^EpCAM(m)^−^EpCAM(t)^+^CK7/8^−^panCK^−^ and CD45^+^EpCAM(m)^+^EpCAM(ic)^−^CK7/8^+^panCK^−^ CHCs were 20.00 (0.25–75.25) cells/mL and 0.00 (0.00–1.00) cells/mL, respectively. After surgery, the medians of the corresponding subpopulations decreased to 8.0 (0.25–26.50) cells/mL and 0.00 (0.00–0.00) cells/mL, respectively.

### 3.3 Association of the CTC and CHC Landscape with Clinical Parameters in Patients with Head and Neck Cancer Prior to Surgery

Another key objective of the study was to investigate the associations between the CTC and CHC spectrum and tumor parameters. To achieve this, we analyzed differences in CTC and CHC counts according to T and N stages (Fig. 6 and Fig. 7).

**Figure 6:**
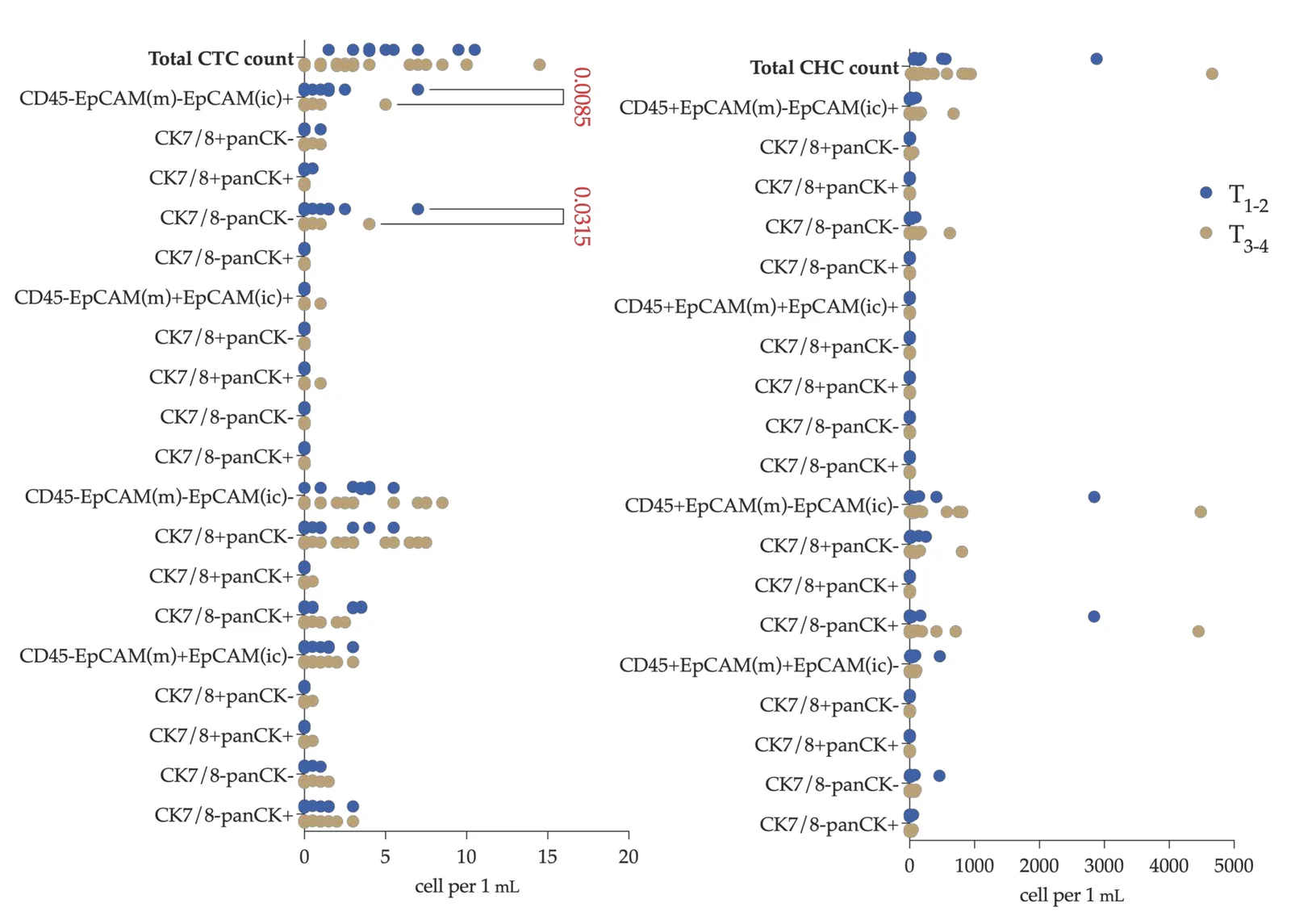
The number of CTCs and circulating hybrid cells (CHCs) in head and neck cancer patients stratified by stage T. The Mann-Whitney test was employed to compare CTC and CHC subpopulations and evaluate *p*-values in patients with head and neck cancer.

Notably, only one CTC population was associated with T stage. The number of CD45^−^EpCAM(m)^−^EpCAM(t)^+^ cells was higher in patients with T1–2 (1.5 (0.25–2.0) cells/mL) compared to those with T3–4 (0.00 (0.00–0.50) cells/mL, *p* = 0.0085); this difference was primarily due to CK7/8^−^panCK^−^ cells (2.00 (0.00–4.00) cells/mL versus 0.00 (0.00–1.00) cells/mL, *p* = 0.0315). 

Analysis of the association between the CTC and CHC spectrum and N stage did not reveal significant differences between patients with and without lymph node involvement (Fig. 7).

**Figure 7:**
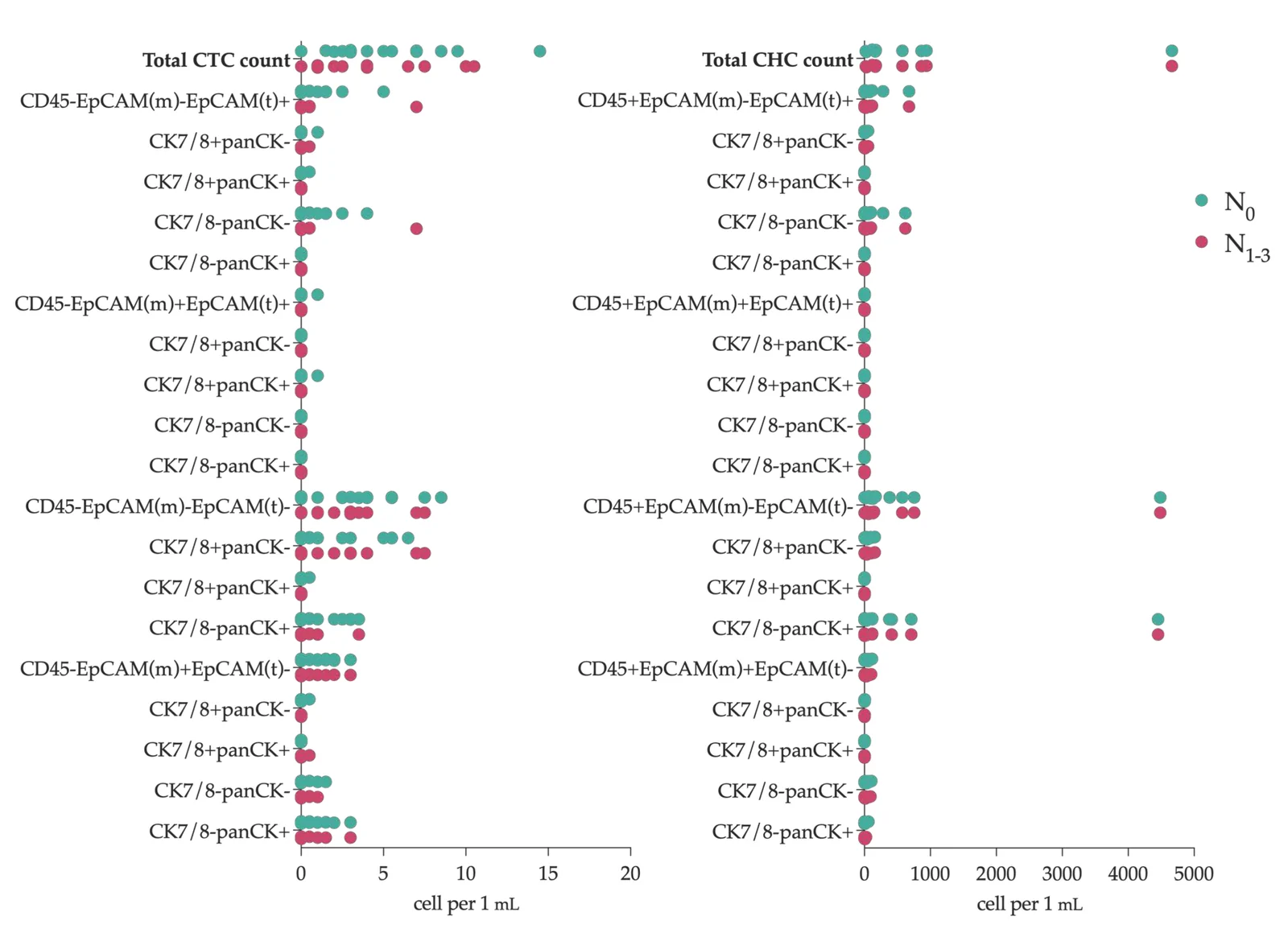
The number of CTCs and CHCs in head and neck cancer patients stratified by stage N. The Mann-Whitney test was employed to compare CTC and CHC subpopulations and evaluate *p*-values in patients with head and neck cancer.

### 3.4 Association of the CTC and CHC Landscape with Recurrence in Patients with Head and Neck Cancer before and after Surgery

In our study, four patients experienced relapse during the 15-month follow-up period. Before surgery, CD45^+^EpCAM(m)^+^EpCAM(ic)^+^CK7/8^+^panCK^−^ CHCs tended to be higher in patients who later developed relapse compared to those without relapse (0.25 (0.00–0.86) cells/mL vs. 0.0 (0.0–0.0) cells/mL, *p* = 0.0523) (Fig. 8). Additionally, we observed a trend toward increased numbers of CD45^+^EpCAM(m)^−^EpCAM(ic)^−^CK7/8^+^panCK^+^ CHCs in patients with relapse prior to surgery (0.25 (0.00–3.50) cells/mL vs. 0.00 (0.00–0.00) cells/mL, *p* = 0.0913).

**Figure 8:**
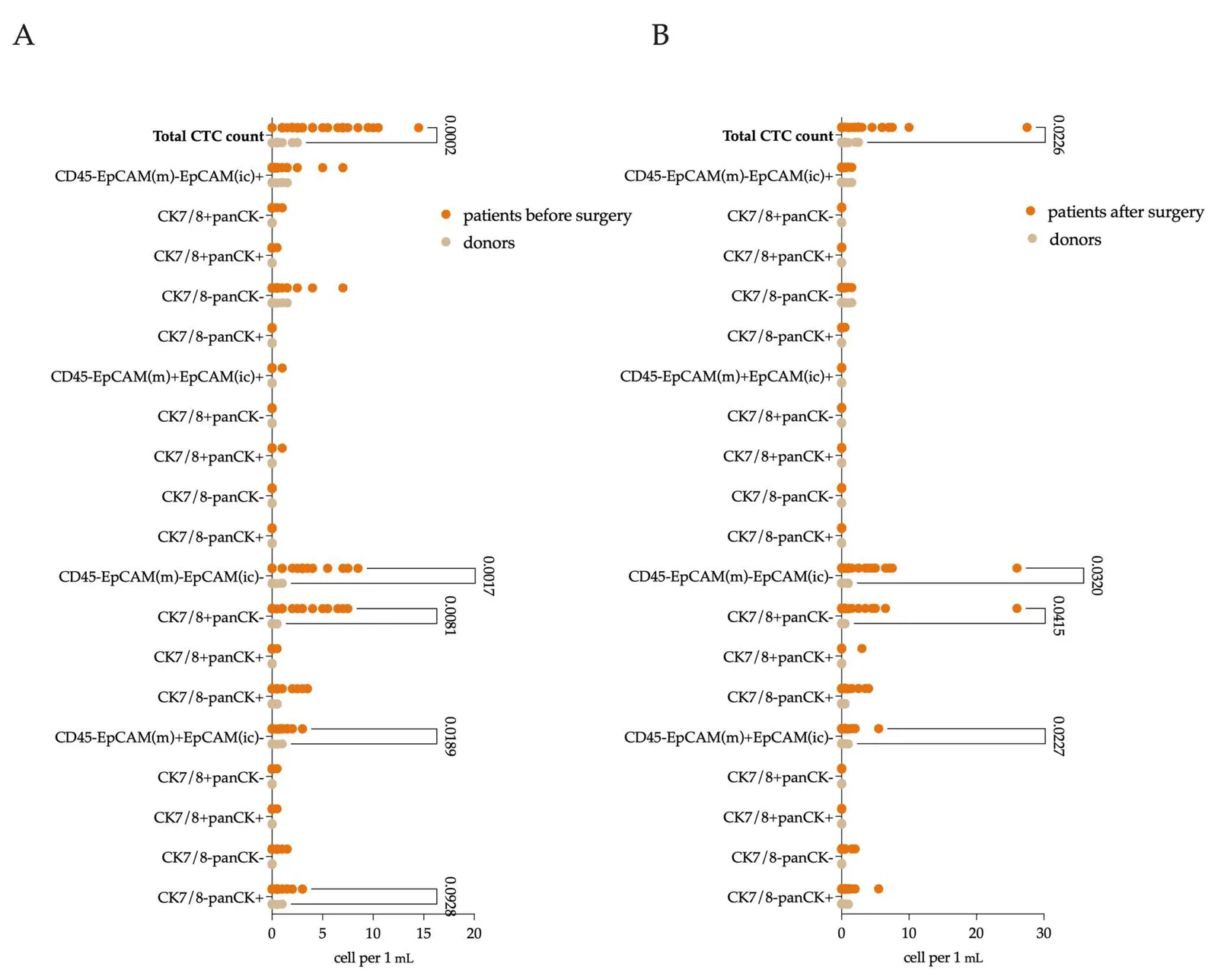
Preoperative CTC and CHC counts in head and neck cancer patients stratified by recurrence status during follow-up.

After surgery in patients who experienced relapse during the observation period, the total number of CHCs tended to decrease (*p* = 0.0774) (Fig. 9). The median post-surgery CHC count in patients with and without recurrence during the follow-up period was 99.5 (42.36–184.50) cells/mL and 29.50 (8.50–67.50) cells/mL, respectively.

**Figure 9:**
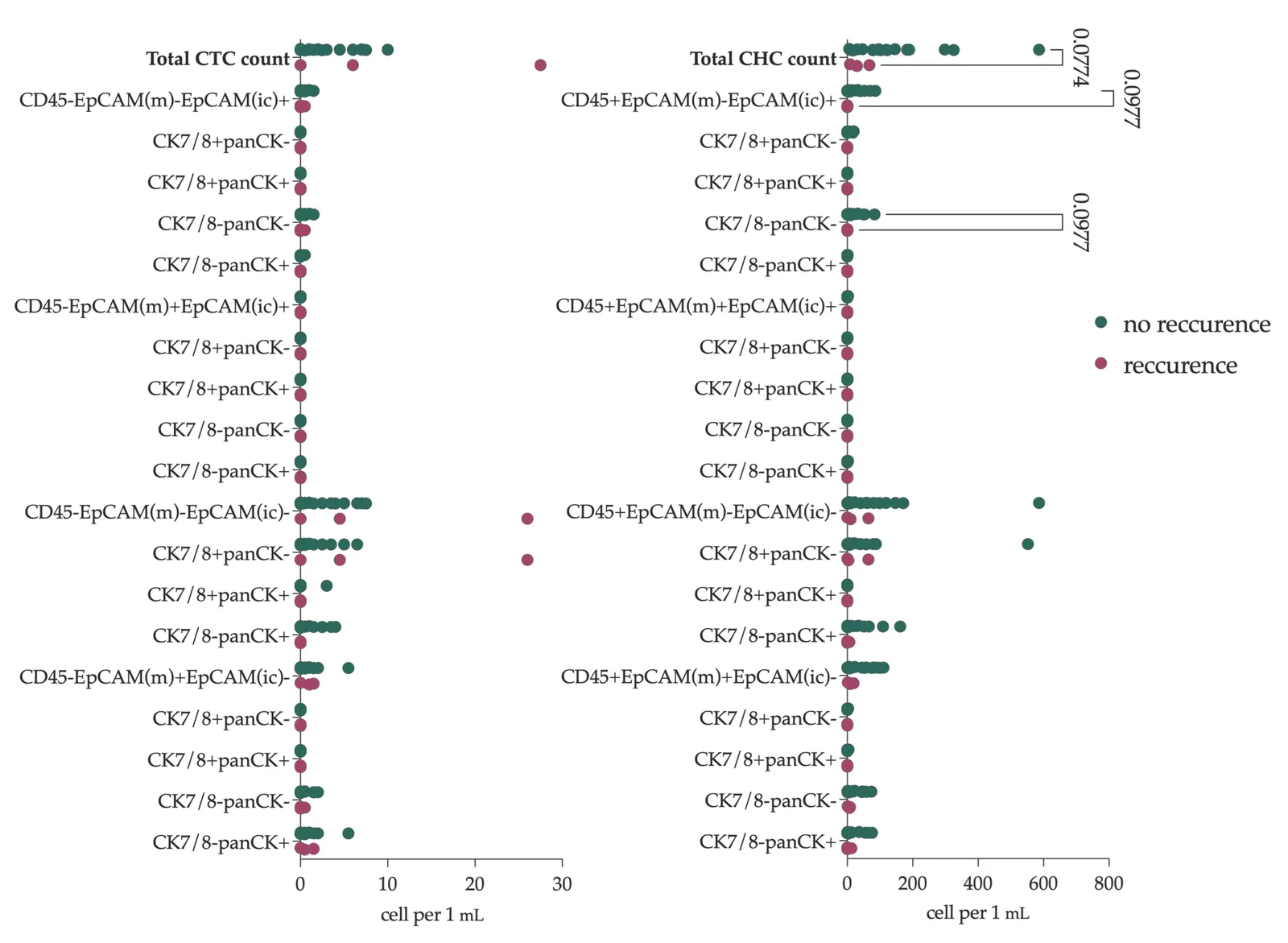
Post-surgery CTC and CHC counts in head and neck cancer patients stratified by recurrence status during follow-up. The Mann-Whitney test was employed to compare CTC and CHC subpopulations and evaluate *p*-values in patients with head and neck cancer.

Thus, it was found that none of the CTC subpopulations showed a significant quantitative decrease in patients after surgical treatment. Furthermore, none of the studied CTC subpopulations were associated with recurrence during the follow-up period. In contrast, among the CHCs, specific subpopulations were identified whose counts significantly decreased following surgical treatment. Notably, among the CHCs detected prior to surgery, the increased count of CD45^+^EpCAM(m)^+^EpCAM(ic)^+^CK7/8^+^panCK^−^ cells showed a trend-level association with recurrence over the 15-month follow-up. Meanwhile, a trend toward decreased CHC counts in peripheral blood after surgery was associated with recurrence (*p* = 0.0774). This observed decrease in CHC count was primarily driven by the CD45^+^EpCAM(m)^−^EpCAM(ic)^+^ population (*p* = 0.0977).

Next, we evaluated the association of the two most promising CHC subpopulations CD45^+^EpCAM(m)^+^EpCAM(ic)^+^CK7/8^+^panCK^−^ detected before surgery and CD45^+^EpCAM(m)^−^EpCAM(ic)^+^ detected after surgery with recurrence-free survival in head and neck cancer patients. To this end, the optimal cutoff for CHC counts was initially determined. The ROC analysis for the CD45^+^EpCAM(m)^+^EpCAM(ic)^+^CK7/8^+^panCK^−^ subpopulation was not significant (*p* = 0.377). However, for the CD45^+^EpCAM(m)^−^EpCAM(ic)^+^ CHCs detected after surgery, a cutoff of 2.50 cells per mL of peripheral blood was identified at a low trend level (*p* = 0.087) with a sensitivity of 100% and specificity of 77.78% (Fig. 10A).

**Figure 10:**
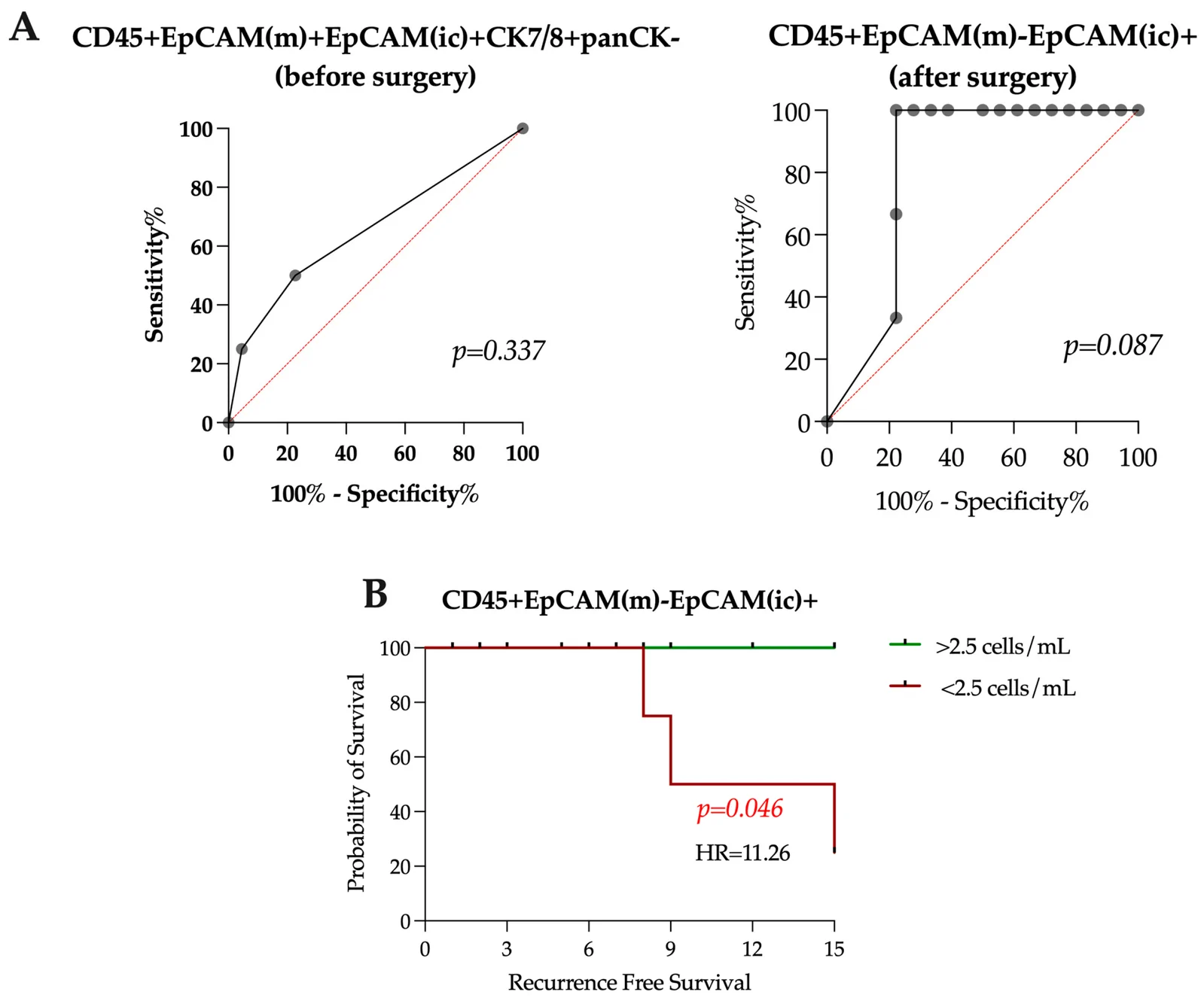
ROC analysis of CD45^+^EpCAM(m)^+^EpCAM(ic)^+^CK7/8^+^panCK^−^ and CD45^+^EpCAM(m)^−^EpCAM(ic)^+^ CHCs (**A**) and recurrence-free survival (**B**) in head and neck cancer patients stratified by CD45^+^EpCAM(m)^−^EpCAM(ic)^+^ CHC counts.

Using the established cutoff for CD45^+^EpCAM(m)^−^EpCAM(ic)^+^ CHC counts, we evaluated recurrence-free survival in head and neck cancer patients over a 15-month follow-up period (Fig. 10B). Patients with a CD45^+^EpCAM(m)^−^EpCAM(ic)^+^ CHC count below 2.5 cells/mL exhibited significantly decreased recurrence-free survival (*p* = 0.046).

To assess the independent association of the established parameter with recurrence-free survival, we performed univariate Cox regression analysis (Table 3). A statistically significant association was found only for the CD45^+^EpCAM(m)^−^EpCAM(ic)^+^ cell population. Specifically, when the cell count was below 2.50 cells/mL, there was an 11.26-fold decrease in recurrence-free survival (HR = 11.26; *p* = 0.0433). No significant associations were observed for T stage or lymph node status: T3–4 versus T1–2 showed HR = 0.91 (95% CI 0.08–19.65; *p* = 0.9398), and N1–3 versus N0 had HR = 2.59 (95% CI 0.22–58.29; *p* = 0.4524).

**Table 3:** Cox regression analysis association of studied clinical parameters with recurrence-free survival.

Factor		HR	95% CI	*p*
CD45^+^EpCAM(m)^−^EpCAM(ic)^+^ CHCs	>2.5 cells/mL	ref		
	<2.5 cells/mL	11.26	1.03–122.20	0.0433
Т	1–2	ref		
	3–4	0.91	0.08–19.65	0.9398
N	0	ref		
	1–3	2.59	0.22–58.29	0.4524

Note: HR: Hazard Ratio; CI: Confidence Interval; circulating hybrid cells (CHCs).

Additionally, we examined correlations between CD45^+^EpCAM(m)^−^EpCAM(ic)^+^ cell counts and T and N stages. A moderate negative correlation was found between lymph node metastasis status (stage N) and hybrid tumor cell count (r = −0.47; *p* = 0.0299), whereas no significant correlation was observed between primary tumor size (stage T) and CHC levels (r = 0.35; *p* = 0.1124).

### 3.5 Relationship between the CTC Landscape and the Primary Tumor Profile in Patients with Head and Neck Cancer

Primarily, to evaluate the relationship between CTC and the primary tumor cells we assessed the number of tumor cell different populations as percent from all tumor cells (Fig. 11).

**Figure 11:**
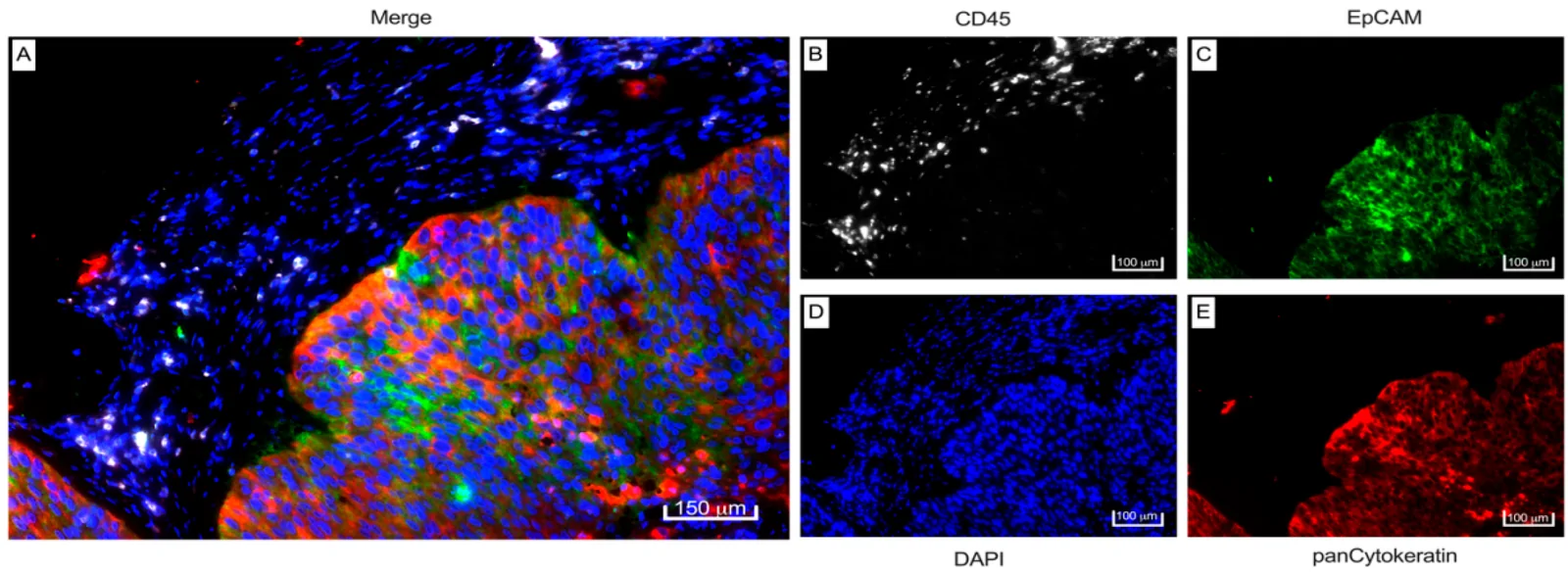
Immunophenotypic analysis of cancer tissue sample stained for CD45, EpCAM, and pan-Cytokeratin using multiplex immunofluorescence. Representative image captured at 400× magnification. (**A**) Tumor tissue showing composite staining of each of the four markers (CD45, EpCAM, pan-cytokeratin, DAPI). (**B**–**E**) Individual images of CD45 (white, B), EpCAM (green, C), DAPI (blue, D) and pan-cytokeratin (red, E).

We analyzed the correlation between the numbers of EpCAM^+^ and panCK^+^ cells in the primary tumor and the counts of EpCAM^+^, panCK^+^, and CK7/8^+^ CTCs (Fig. 12). 

**Figure 12:**
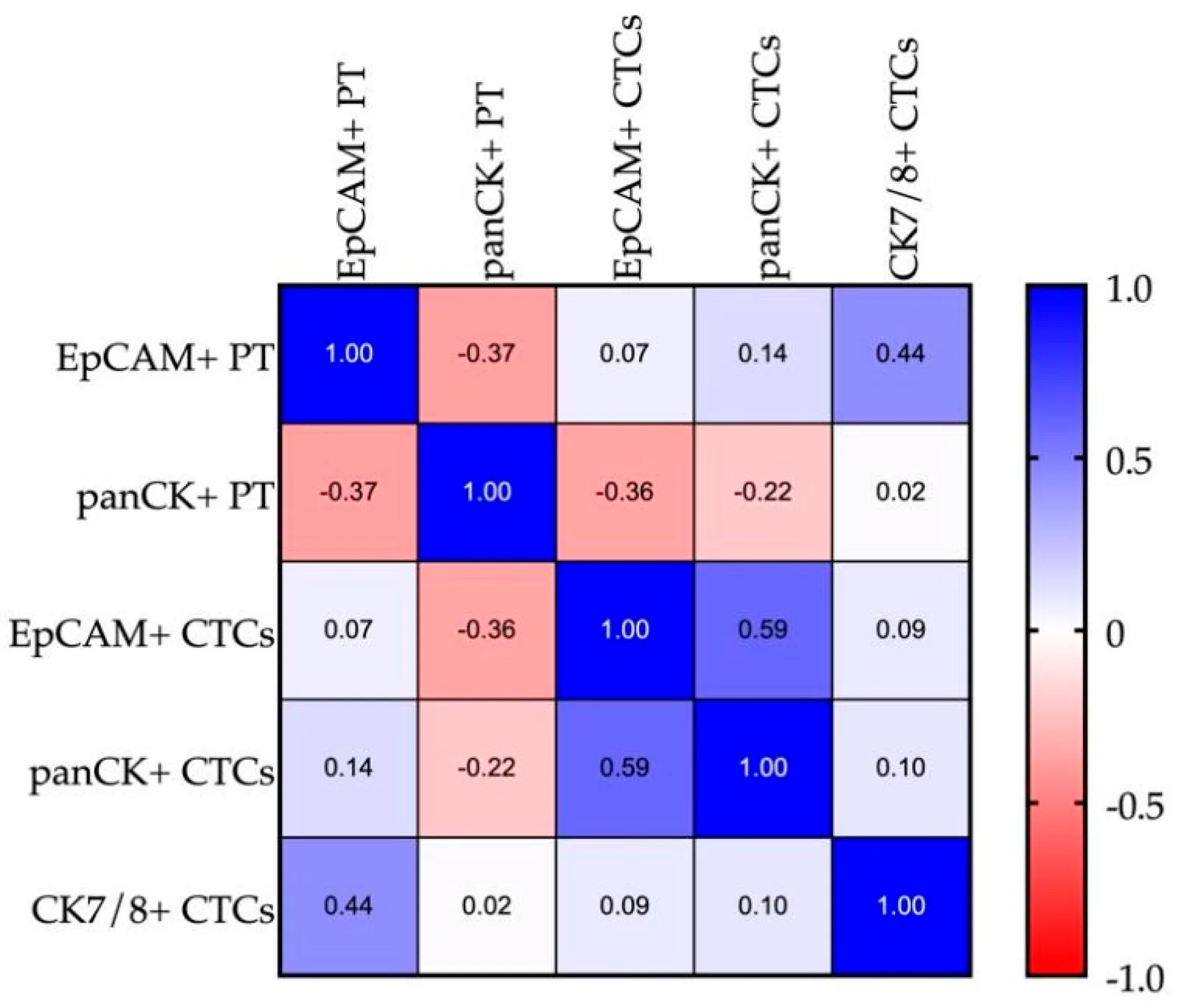
Correlation analysis between the numbers of EpCAM^+^ and panCK^+^ cells in the primary tumor and the counts of EpCAM^+^, panCK^+^, and CK7/8^+^ CTCs. Footnote: PT–primary tumor.

No significant associations were observed between the tumor cell populations and their corresponding CTCs. However, the numbers of EpCAM^+^ and panCK^+^ CTCs were positively correlated (r^2^ = 0.595, *p* = 0.001), whereas no correlation was found between panCK^+^ and CK7/8^+^ CTCs.

### 3.6 Comparison of the CHC Landscape in Healthy Donors and Head and Neck Cancer Patients

To clarify the potential origin of CHCs, we applied the same monoclonal antibody panel to peripheral blood cells from healthy donors. Fig. 13 presents the heterogeneity of CHCs in the blood of healthy donors based on EpCAM and cytokeratin expression.

**Figure 13:**
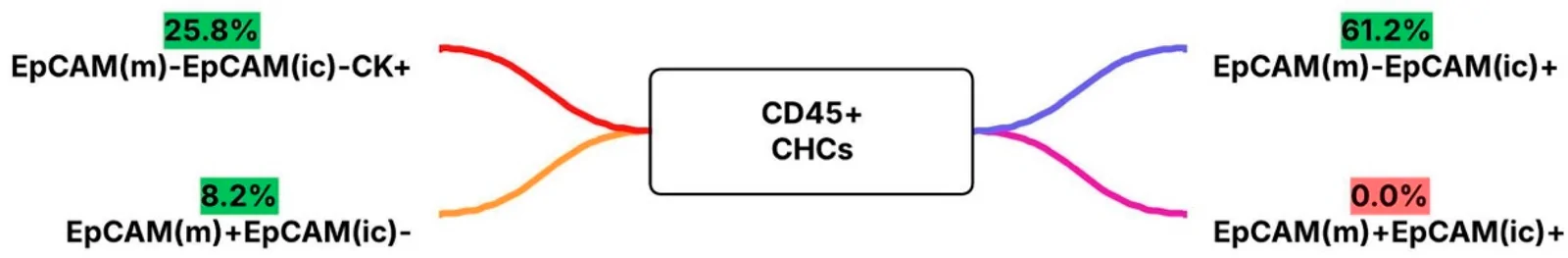
The median proportion of different CHC subpopulations in healthy donors.

The median of CHC count in healthy donors was 43.25 (32.00–80.13) cells/mL, which was significantly lower compared to the pre-surgery patients 170.50 (79.00–557.88) cells/mL (*p* = 0.0004) and did not differ significantly from post-surgery patients: 93.00 (29.75–164.25) cells/mL (Fig. 14). The count of main CHCs subpopulations in three studied groups presented in Table 4.

**Table 4:** CHC counts in peripheral blood of head and neck cancer patients (before and after surgery) and healthy donors.

CHC Phenotype	CHCs count in Patients before Surgery, Cell/mL (1)	CHCs Count in Patients after Surgery, Cell/mL (2)	Healthy Donors, Cell/mL (3)	*p*-Value
EpCAM(m)^−^EpCAM(ic)^−^, median (IQR)	70.25 (26.50–246.80)	22.50 (5.50–89.75)	15.50 (7.00–25.63)	p_1–3_ = 0.0014
EpCAM(m)^−^EpCAM(ic)^+^, median (IQR)	20.00 (1.25–79.00)	8.00 (0.25–29.25)	21.25 (12.13–51.63)	
EpCAM(m)^+^EpCAM(ic)^−^, median (IQR)	34.00 (8.75–67.38)	19.00 (5.00–66.50)	3.00 (2.13–6.75)	p_1–3_ = 0.0011 p_2–3_ = 0.0324
EpCAM(m)^+^EpCAM(ic)^+^, median (IQR)	0.00 (0.00–0.50)	0.00 (0.00–0.50)	0.00 (0.00–0.25)	

Note: circulating hybrid cells (CHCs).

**Figure 14:**
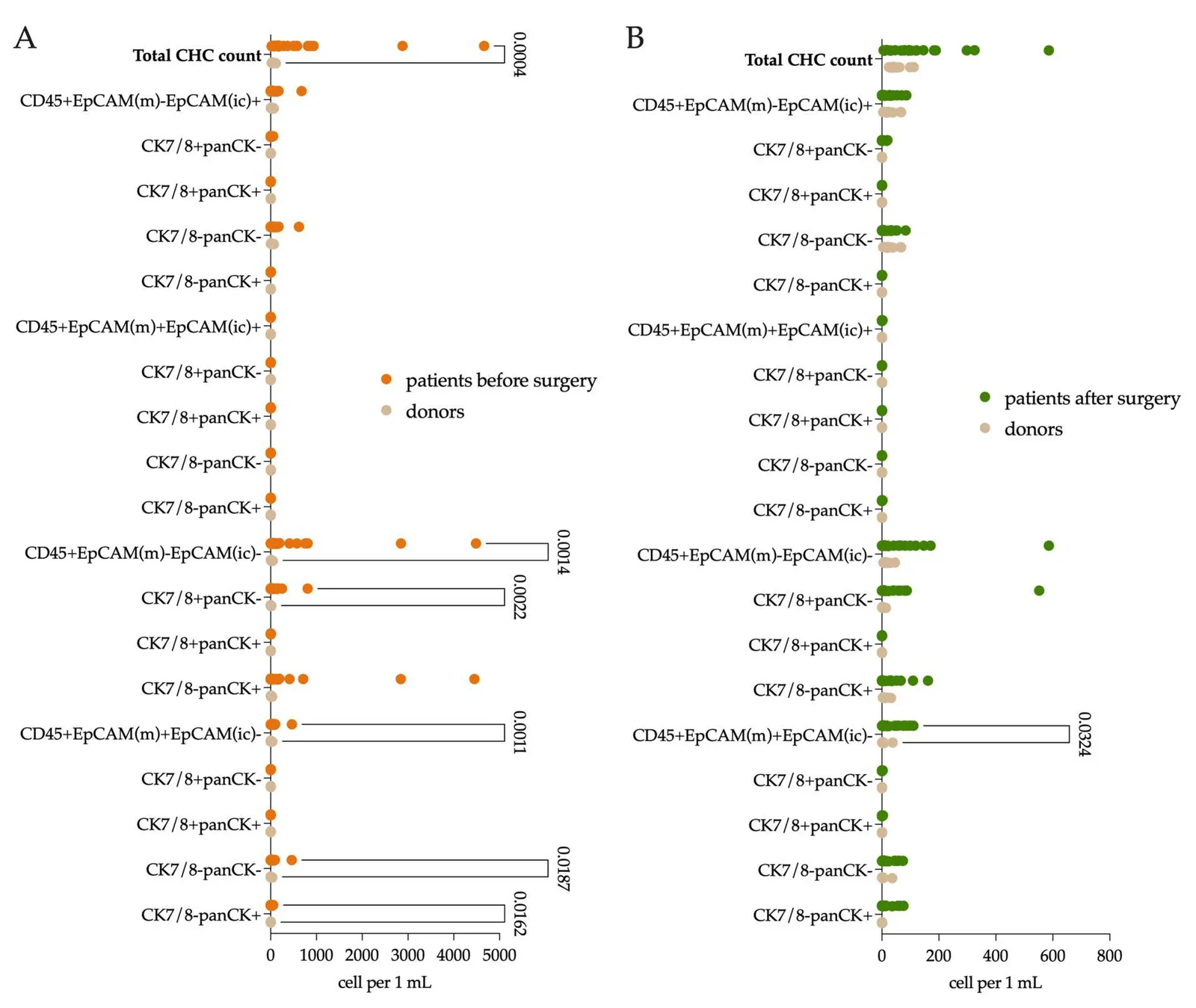
The number of CHCs in healthy donors and head and neck cancer patients before (**A**) and after (**B**) surgery. The Mann-Whitney test was employed to compare CTC and CHC subpopulations and evaluate *p*-values in patients with head and neck cancer and healthy donors.

Patients before surgery exhibited significantly higher total CHC counts compared to healthy donors (*p* = 0.0004). This increase was driven by specific CHC subpopulations. Notably, the number of CD45^+^EpCAM(m)^−^EpCAM(ic)^−^CK7/8^+^panCK^−^ cells was elevated before surgery (*p* = 0.0022) (Fig. 14A), but following surgery, these differences in their counts disappeared, showing no difference between patients and donors (*p* > 0.05) (Fig. 14B). The median counts of CD45^+^EpCAM(m)^−^EpCAM(ic)^−^CK7/8^+^panCK^−^ cells in healthy donors and patients before and after surgery were 2.50 (1.75–9.50) cells/mL, 26.25 (7.50–45.25) cells/mL, and 9.00 (3.25–49.50) cells/mL, respectively. A similar pattern was observed for the CD45^+^EpCAM(m)^+^EpCAM(ic)^−^CK7/8^−^panCK^−^ and CD45^+^EpCAM(m)^+^EpCAM(ic)^−^CK7/8^−^panCK^+^ subpopulations (Fig. 14). The counts of these cells in patients before surgery were significantly higher than those in donors (*p* = 0.0187 and *p* = 0.0162, respectively). Median counts of CD45^+^EpCAM(m)^+^EpCAM(ic)^−^CK7/8^−^panCK^−^ cells in donors, patients before surgery, and patients after surgery were 2.00 (1.63–5.38), 15.50 (3.38–44.75), and 8.50 (1.50–45.00) cells/mL, respectively. For CD45^+^EpCAM(m)^+^EpCAM(ic)^−^CK7/8^−^panCK^+^ cells, median counts were 0.50 (0.38–1.13), 5.00 (0.88–21.88), and 1.50 (0.25–13.25) cells/mL, respectively. After surgery, no significant differences in the numbers of these subpopulations were observed between patients and donors (*p* > 0.05). However, the total number of the CD45^+^EpCAM(m)^+^EpCAM(ic)^−^ subpopulation in patients after surgery remained significantly higher than in donors (*p* = 0.0324).

## 4 Discussion

Recent studies demonstrate that surgical intervention can significantly impact both the quantity and characteristics of classical CTCs. In the early postoperative period, a sharp increase in CTC counts is often observed, which can be explained by a combination of factors: mechanical release of tumor cells during surgery, increased vascular permeability due to the inflammatory response, and the removal of the primary tumor’s suppressive effect that had previously restrained the dissemination of these cells. This phenomenon has been particularly well studied in breast and colorectal cancers, where a marked increase in CTC counts within the first 24–48 h after surgery correlates with poorer long-term treatment outcomes for patients [25,26,27]. Clinical observations confirm the prognostic significance of postoperative dynamics in CTC counts [28]. For example, in a case report by Wu et al. (2016), an exceptionally high level of CTCs—up to 1400 cells per 7.5 mL—was documented prior to rapid disease recurrence and a fatal outcome within two months [29]. 

We observed that surgical resection significantly reduced the abundance of CHCs but did not affect the overall population of classical CD45^−^ CTCs. This result leads us to reject the null hypothesis that surgical intervention has no impact on these circulating cell populations. Furthermore, our study provides new insights into the phenotypic landscape of circulating cells with epithelial features in patients with head and neck cancer. By distinguishing between CTCs and CHCs and assessing epithelial features using four molecular markers (EpCAM(m), EpCAM(ic), CK7/8, panCK), we reveal a high prevalence of CHCs in comparison with CTC counterparts. Indeed, CHCs were detected in all patients and outnumbered CTCs by more than forty-fold. 

The phenotypic heterogeneity observed in both CD45^−^ and CD45^+^ compartments highlights the dynamic interplay between epithelial and mesenchymal programs. Subpopulations lacking membrane EpCAM expression but retaining intracellular EpCAM or cytokeratin expression provide strong evidence of ongoing EMT. In particular, CD45^−^EpCAM(m)^−^EpCAM(ic)^+^ CTCs, which predominantly lack cytokeratins expression, may represent a more mesenchymal-like subset of CTCs, consistent with an advanced EMT phenotype. Intriguingly, parallel subpopulations were also found within CHCs, and the similarity in distribution patterns between CD45^+^ and CD45^−^ phenotypes indirectly support the tumor origin of at least a portion of the CHCs.

Numerous studies indicate that surgery can selectively promote the survival of the most aggressive CTC subsets, particularly those exhibiting EMT features [30,31]. In our study, surgical resection significantly reduced the abundance of CHCs but had no effect on the overall population of CTCs. At the subpopulation level, specific CD45^+^ phenotypes—such as EpCAM(m)^−^EpCAM(ic)^+^CK7/8^−^panCK^−^ and EpCAM(m)^+^EpCAM(ic)^−^CK7/8^+^panCK^−^—exhibited a marked postoperative decrease, whereas CD45^−^ subpopulations remained largely stable. These findings suggest that CHCs are more dynamically influenced by tumor removal, whereas classical CTCs may persist independently of the local tumor burden. The sustained presence of CTCs may reflect minimal residual disease or alternative dissemination routes not entirely addressed by surgical excision. 

Associations with clinicopathological features further strengthen the biological relevance of CTC heterogeneity. The abundance of CD45^−^EpCAM(m)^−^EpCAM(ic)^+^ cells correlated with earlier T stage. This phenomenon could be explained by the greater intratumoral heterogeneity of advanced tumors (T_3_-T_4_), which results in the production of a wider spectrum of diverse CTC phenotypes. Consequently, this could result in a more uniform distribution of the CTC pool rather than enrichment of specific subpopulations.

Analysis of blood cells from healthy donors revealed the presence of various CHC subpopulations, although in significantly lower numbers compared to untreated patients. This indicates extreme heterogeneity of CHCs and likely different origins, both tumor-derived and non-tumor-derived.

The presence of CD45^+^EpCAM(m)^−^EpCAM(ic)^+^ CHCs in healthy donors at levels comparable to pre-surgery patients—21.25 (12.13–51.63) cells/mL versus 20.00 (1.25–79.00) cells/mL—supports their non-tumor origin. Notably, postoperative depletion of this CHC subpopulation was associated with poor clinical outcomes. One hypothesis is that these hybrid CHCs might be involved in immune surveillance processes, potentially detecting, and presenting cancer cell fragments to activate systemic immunity. The elevated CHC levels seen in healthy individuals and preoperative patients might reflect such baseline activity. Surgery causes tissue damage and releases cellular debris, including cancer cells, into circulation; probably, in immunocompetent patients, CHC levels appear to remain relatively stable postoperatively, which could correspond to ongoing clearance and tumor cell targeting activities. Maintaining CHC counts above 2.5 cells/mL after surgery was associated with better clinical outcomes, which might indicate a more effective immune environment in these cases. Conversely, a postoperative drop below this threshold could suggest impaired immune surveillance, potentially allowing residual cancer cells to persist and contribute to relapse. Since classical CTC counts did not decrease after surgery, it remains possible that circulating cells with tumor-promoting potential persist in the blood of some patients. Overall, these findings warrant further investigation into the functional role of CHCs, which may serve as valuable prognostic markers of tumor progression in head and neck cancer.

Interestingly, comparison of CHC counts between healthy donors and patients before surgery identified distinct subpopulations—CD45^+^EpCAM(m)^−^EpCAM(ic)^−^ and CD45^+^EpCAM(m)^+^EpCAM(ic)^−^—that were elevated in cancer patients and decreased following tumor resection. Although these cells were also present in healthy donors at lower levels, their increased abundance in patients suggests a possible tumor origin for a portion of these cells.

Although our study involved a limited number of patients, the use of appropriate statistical methods supports the significance of the main findings, while associations approaching statistical significance (*p* = 0.05–0.1), which correspond to preliminary data, need further investigation. Collectively, our findings expand the conceptual framework of CTC biology in head and neck cancer by demonstrating that CHCs constitute a major, clinically relevant compartment with distinct dynamics and associations compared to classical CTCs. The identification of specific subpopulations linked to stage and recurrence highlights the need for integrative monitoring strategies that go beyond enumeration of bulk CTCs. 

Future research should investigate the functional roles of hybrid CHCs in larger cohorts of head and neck cancer patients, including their involvement in metastatic dissemination and immune evasion, and evaluate their potential as biomarkers for risk stratification and personalized therapeutic guidance.

## 5 Conclusion

This study reveals that the circulating cell landscape in head and neck cancer is dominated by CHCs, which outnumber classical CTCs by over forty-fold. The differential response to surgical intervention—with marked depletion of specific CHC subpopulations but persistence of CTCs—suggests distinct biological origins and prognostic implications. Notably, postoperative CHC counts above 2.5 cells/mL were associated with favorable clinical outcomes, which could suggest a possible role of these cells in tumor surveillance.

Our findings provide a framework for integrative monitoring of both classical CTCs and hybrid CHCs in head and neck cancer. These distinct circulating populations may offer complementary prognostic information regarding tumor burden, EMT status, and immune competence. Future studies are warranted to elucidate the functional properties of CHC subpopulations and to evaluate their clinical utility for risk stratification and therapeutic decision-making in head and neck cancer.

## Figures and Tables

**Figure A1:**
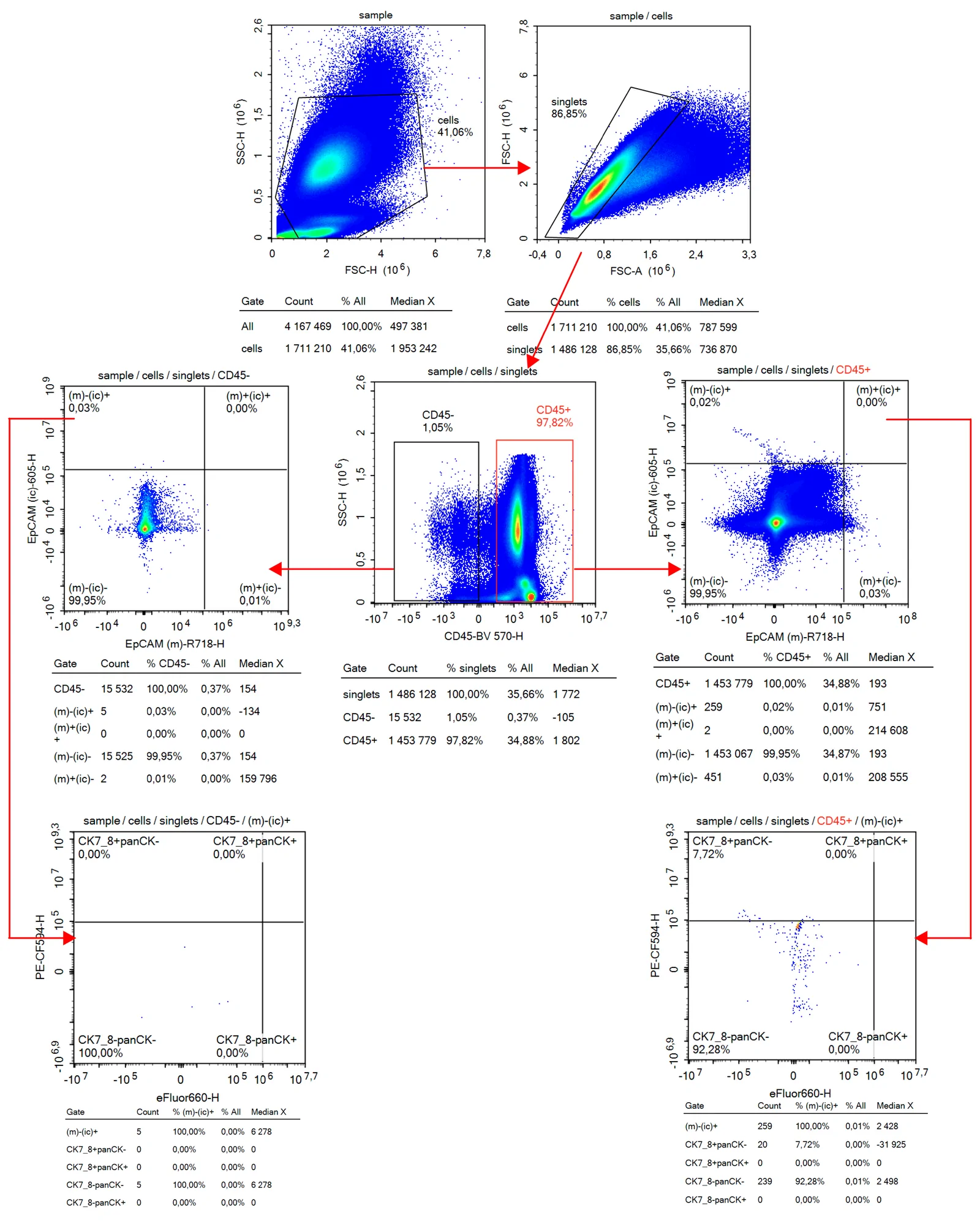
**The gating strategy of CD45^−^ CTCs and CD45^+^ circulating hybrid cells (CHCs) in patients with head and neck cancer.** A hierarchical gating strategy was applied. Following the exclusion of debris (‘Cells’ gate) and doublets (‘Singlets’ gate), total cells were separated into CD45-negative and CD45-positive populations. Epithelial subsets were then identified within each of these fractions based on two criteria: (1) the expression of EpCAM (both membrane and intracellular), and (2) the expression of cytokeratin 7/8 and pan-cytokeratin.

## Data Availability

Inquiries can be directed to the corresponding author.
